# Supplementary material for: Childhood Trauma and Adult Somatic Symptoms
Source: Psychosom Med. 2023 Apr 26;85(5):408–16. doi: 10.1097/PSY.0000000000001208 (PMC10241439; doi:10.1097/PSY.0000000000001208)
Supplement: Supplementary file 1 [file psymed-85-408-s001.docx]

**Supplementary Tables**

**Table S1**

*Bivariate correlations between childhood trauma types.*

| Variable | 1. | 2. | 3. | 4. | 5. |
| --- | --- | --- | --- | --- | --- |
| 1. Physical Abuse | - | .47^*^ | .39^*^ | .45^*^ | .37^*^ |
| 2. Emotional Abuse | .56^*^ | - | .42^*^ | .59^*^ | .72^*^ |
| 3. Sexual Abuse | .30^*^ | .41^*^ | - | .42^*^ | .33^*^ |
| 4. Physical Neglect | .31^*^ | .57^*^ | .34^*^ | - | .61^*^ |
| 5. Emotional Neglect | .37^*^ | .68^*^ | .31^*^ | .55^*^ | - |

*Note*. **p* < .01. *N* = 406 above the diagonal, representing the cross-sectional sample, and *N* = 290 below the diagonal, representing the EMA sample.

**Table S2**

*Multilevel model of momentary physical discomfort by childhood trauma (CTQ) scores (Model 1) and subtypes (Model 2).*

| Predictors | *Estimate* | *SE* | *t* | *p* |  |
| --- | --- | --- | --- | --- | --- |
| *Model 1* |  |  |  |  |  |
| Intercept  Gender | 22.00  7.88 | 5.11  2.97 | 4.31  2.66 | **<.001**  **.008** |  |
| Age | -0.11 | 0.08 | -1.42 | .157 |  |
| Relationship status | 1.61 | 2.30 | 0.70 | .485 |  |
| Education level | 2.61 | 3.26 | 0.80 | .424 |  |
| Childhood trauma^1^ | 0.34 | 0.09 | 3.80 | **<.001** |  |
| *Model 2* |  |  |  |  |  |
| Intercept | 22.63 | 5.12 | 4.42 | **<.001** |  |
| Gender | 7.25 | 3.03 | 2.39 | **.018** |  |
| Age | -0.12 | 0.08 | -1.17 | .140 |  |
| Relationship status | 1.82 | 2.33 | 0.78 | .434 |  |
| Education level | 2.60 | 3.28 | 0.79 | .428 |  |
| Physical Abuse^1^ | -0.48 | 0.59 | -0.81 | .421 |  |
| Emotional Abuse^1^ | 0.76 | 0.43 | 1.76 | .080 |  |
| Sexual Abuse^1^ | 0.42 | 0.41 | 1.01 | .315 |  |
| Physical Neglect^1^ | 0.05 | 0.58 | 0.08 | .937 |  |
| Emotional Neglect^1^ | 0.33 | 0.33 | 1.00 | .319 |  |

*Note.* *N*= 290. ^1^= Variable was centered. CTQ = childhood trauma questionnaire. SE= Standard Error. Gender: 0= men, 1= women. Relationship status: 0= single, 1= has a partner. Educational level: 0 = up to secondary education, 1 = tertiary education. Dependent variable: physical discomfort (scale 0-100). Degrees of freedom were 290 and 291 for emotional abuse in model 2. Significant estimates are provided in bold.
