# Supplementary material for: Childhood Trauma and Adult Somatic Symptoms
Source: Psychosom Med. 2023 Apr 26;85(5):408–16. doi: 10.1097/PSY.0000000000001208 (PMC10241439; doi:10.1097/PSY.0000000000001208)
Supplement: Supplementary file 2 [file psymed-85-408-s002.docx]

**Childhood Trauma and Adult Somatic Symptoms**

Hanna Eilers (MSc), Marije aan het Rot (PhD, ORCID: 0000-0001-6761-7513), Bertus Jeronimus (PhD, ORCID: 0000-0003-2826-4537)

Department of Psychology, University of Groningen

hanna_eilers@gmx.de, m.aan.het.rot@rug.nl, b.f.jeronimus@rug.nl

**Address for Correspondence**

Marije aan het Rot, University of Groningen, Department of Psychology, Grote Kruisstraat 2/1, 9712 TS Groningen, The Netherlands, email: m.aan.het.rot@rug.nl

Number of Tables: 2 Number of figures: 1, Number of Supplemental Digital Content files: 1

**Article Editor:** Harald Guendel

Declaration of interest: none

Funding: This study used data collected in the HowNutsAreTheDutch project. BFJ was supported by a VENI grant (016.Veni.195.405) from the Talent Programme of the Netherlands Organization of Scientific Research.

**Abstract**

**Objective:** Childhood trauma is a key public health risk factor for developing physical illness. This study examined how childhood abuse and neglect predict adult somatic symptoms assessed retrospectively and in real-time. **Methods:** Participants in the HowNutsAreTheDutch (HND) project completed the Childhood Trauma Questionnaire Short Form (CTQ-SF) and, to assess somatic symptoms, the cross-sectional Patient Health Questionnaire (PHQ-15; *N* = 406) or a longitudinal ecological momentary assessment (EMA) protocol which included responding to an item on physical discomfort 3 times/day for 30 days (*N* = 290). **Results:** As hypothesized, childhood trauma was positively associated with adult somatic symptoms assessed using the PHQ-15 (*d* = 0.30) and assessed using the EMA item (*d* = 0.31), also after adjustment for age, gender, educational level, and relationship status. Longitudinally, we also explored whether interpersonal context moderated abuse outcomes, and observed that social company dampened momentary physical discomfort among people with a history of more physical neglect (*d* = 0.04). **Conclusion***:* Childhood trauma, especially emotional and sexual abuse, predict specific adult somatic symptoms. Future work may examine how symptom expression is influenced by the social lives of traumatized individuals.

**Keywords**: child abuse, child neglect, child maltreatment, physical health, interpersonal context, ecological momentary assessment.

**List of abbreviations**

ANCOVA = Analysis of Covariance

CTQ = Childhood Trauma Questionnaire

CTQ-SF = Childhood Trauma Questionnaire Short Form

EMA = Ecological Momentary Assessment

HND = HowNutsAreTheDutch

HPA = hypothalamus-pituitary-adrenal

PHQ = Patient Health Questionnaire

VAS = Visual Analogue Scale

**Introduction**

Childhood trauma emerges when children are overwhelmed by abuse, neglect, and other exploitations that harm health, development, and dignity (1). Almost 40% of humans are exposed to adverse childhood experiences (2). European community estimates of emotional (~18%) and physical (~16%) neglect and emotional (~16%), physical (~23%), and sexual (~10%) abuse also indicate that trauma is a key public health issue (3).

Childhood trauma not only harms health and well-being in childhood but also increases the risk of adult mental and physical health problems and, more broadly, unhappiness (4,5). Childhood trauma has been associated with a heightened risk of developing all leading causes of death and disability worldwide, for example, heart disease, cancer, stroke, and diabetes, and depression and suicidality (6). Further, childhood trauma has been associated with adult reports of specific somatic symptoms such as chronic pain and headache (7,8). Furthermore, although many studies have focused on the impact of physical and sexual abuse, emotional abuse and neglect have been associated with somatic symptoms as well (9).

In childhood trauma studies, trauma severity and the risk of physical health conditions typically follow a dose-response relationship (4), with odds that can increase fourfold (10). A prominent theory to connect childhood trauma to adult physical health problems is a stress-driven hypothalamus-pituitary-adrenal (HPA) dysregulation that impedes the body’s ability to maintain homeostasis in response to changing conditions (allostasis), resulting in toxic stress and hormonal disturbances (11,12). This risk is relatively large in children as their neural, endocrine, and immune systems show rapid development and are very sensitive (13). HPA adaptation to adverse environments may contribute to allostatic overload via bodily wear and tear that increase the risk of poor health and well-being decades later (11,14).

Most childhood trauma involves a parent (~80%) or close caregiver as perpetrator (e.g., relative or babysitter (15)). This alters children’s expectations about interpersonal relationships, fosters insecurity and distrust (16,17), and causes them to develop negative cognitive schemas about themselves in relation to others (18). This may interfere with the formation and maintenance of positive interpersonal relationships (19). However, having such positive relationships is thought to play a key role in facilitating post-trauma adjustment and well-being, including mental and physical health (20,21). This is known as the stress buffering hypothesis (22). Ironically then, even though traumatized individuals in particular could benefit from positive social interactions, they are more likely to struggle to establish the interpersonal relationships that can help reduce their physical complaints. Conversely, traumatized individuals who are able to form positive relationships with others and have positive social interactions may therefore be less likely to report somatic symptoms.

**The present study**

We conducted the present study in light of several observations made when reviewing the literature. First, previous studies of childhood trauma increasing the risk of adult physical illness often focused on specific medical diagnoses (e.g., cardiovascular disease (23)). In comparison, we examine this association using common somatic symptoms at a population level. These physical complaints may or may not match the description of a diagnosis, medical syndrome, or organic disease, but often cause considerable distress, disability, burden, or medical consultation (10,24). This lower threshold makes our model sensitive and inclusive of what is known as “bodily distress” or “medically unexplained symptoms,” considered an important component of the global burden of disease.

Second, while most previous studies focused on childhood sexual and physical abuse (25,26), we also cover emotional and physical neglect and emotional abuse, which can also have a detrimental impact on body, brain, and mind (5). We examined overall childhood trauma as well as these five trauma types and their specific and cumulative associations with adult somatic symptoms, while accounting for their frequent co-occurrences (4). Only 3 of 244 studies in a recent meta-analytic review had measured all types of abuse and neglect (2).

Third, most past research has used retrospective self-reports of somatic symptoms which may result in biased estimates (e.g., peak- and end-effect bias (27,28)). In contrast, ecological momentary assessment (EMA) serves to repeatedly sample experiences in naturalistic settings, which reduces recall bias and increases ecological validity (29). These rich longitudinal data were available in addition to retrospective data.

The present study thus expands the literature on the association of childhood trauma with adult somatic symptoms in multiple ways. Unlike most previous studies, we used a population sample derived from the HowNutsAreTheDutch project (HND; see below). Besides, we used multiple methods for assessing adult somatic symptoms, i.e., a retrospective symptom checklist of common complaints and a 30-day EMA study of physical discomfort.

We hypothesized that childhood trauma would be positively associated with experiencing somatic symptoms in adulthood assessed using retrospectively and in near real-time, using EMA. We also explored whether interpersonal context (being in the company of others vs. being alone) moderated adult trauma survivors’ momentary levels of physical discomfort. Such within-person dynamic patterns in somatic symptoms (27) were predicted given the interpersonal roots of childhood abuse and neglect (3). On the one hand, the social company of participants with childhood trauma could include perpetrators of the trauma, as well as more recently acquainted individuals whom traumatized participants might find it difficult to establish a relationship with. Consequently, somatic symptoms might be more common when people who experienced childhood trauma were in company of others than when they were alone. On the other hand, and in line with the aforementioned stress buffering hypothesis, we considered it possible that adult participants with childhood trauma would report fewer somatic symptoms when in social company.

Overall, more insight into the association between childhood trauma and adult somatic symptoms might help clarify the origin of these symptoms and offer new information on how to reduce them or prevent development into physical disease.

**Method**

**Participants**

Data were derived from the HND crowdsourcing study (30,31). We selected adults (aged ≥ 18 years) who completed the Childhood Trauma Questionnaire (CTQ) and who completed the 24-h time frame version of the Patient Health Questionnaire (PHQ-15, *n* = 406) or ≥ 65% of the EMA measurements (*n* = 290; measures described below). There were 54 participants who completed all three measures.

We restricted ourselves to participants who completed at least 65% of the EMA entries because it was the minimum number of assessments to derive sufficient statistical power for reliable individual models and participant feedback and it was therefore in line with other HND studies (30). The EMA study required participants who had smartphones, who did not anticipate a major disruption of daily routines or shift work within the 30-day study period, and who approved the use of their anonymous data for research purposes.

**Procedure**

Dutch citizens were invited to participate in the HND project at www.HoeGekIs.nl by means of radio broadcasts, television, newspapers, magazines, podium discussions, and social media. Participants had to register on the website and create an account and could then complete multiple mental health questionnaires (or topical modules) and/or take part in the EMA study. The HND platform was launched on December 19^th^ 2013 and the dataset for the present study was extracted on December 19^th^ 2018.

The first four questionnaires pertained socio-demographic variables (“start module” and “living situation”) and affect/mood and well-being. After completing these, all other modules became available, including the somatic symptoms and childhood trauma modules. These could be completed in any order. Hence, while 1595 participants completed the CTQ (childhood trauma module) and 1848 participants completed the 24-h PHQ-15 (somatic symptoms module), 406 participants completed both modules.

The EMA study was launched on May 22^nd^ 2014 and the dataset for the present study was also extracted on December 19^th^ 2018. In the EMA study, participants monitored themselves thrice daily over 30 consecutive days by means of an electronic diary (yielding a maximum of 90 assessments per participant). There were 644 individuals who completed ≥ 65% of the EMA measurements (*M* = 75, *SD* = 7.44, range = 59-90) of whom 290 also completed the CTQ. Diary invitations were sent as text messages (using SMS) at equidistant time points (6-hour intervals), to capture the morning, afternoon, and evening, according to participants’ sleep-wake cycles (e.g., 8:30 a.m., 2:30 p.m., and 8:30 p.m.). Participants could complete the questionnaire within one hour after the invitation. The HND study was approved by the Medical Ethical Committee of the University Medical Center Groningen. All details and procedures are described in the baseline papers (30,31).

**Measures**

***Childhood trauma***

The Childhood Trauma Questionnaire-Short Form (CTQ-SF; 32) examines recollections of five dimensions of childhood trauma with 5 items each: physical, emotional, and sexual abuse, and physical and emotional neglect. The frequency of each item is scored on a 5-point Likert (1 = *never true,* 2 = *rarely true,* 3 = *sometimes true,* 4 = *often true,* 5 = *very often true*). Clinical cut-off values are given for trauma severity levels: none or minimal (score ≤ 36), low to moderate (37-51), moderate to severe (52-68), and severe to extreme trauma (≥ 69). Although a retrospective questionnaire on childhood trauma may suffer from both under- and overreporting, previous work has found different scores for groups know to vary on childhood trauma, thereby demonstrating known-groups validity (33). Besides, the CTQ has shown convergence with a childhood trauma interview, indicating similarity in self-rated versus clinician-rated reports of trauma (34) and with data from knowledgeable informants (32).

The present study showed good to excellent internal consistency for Physical Abuse (.84), Emotional Abuse (.88) and Sexual Abuse (.88), and Emotional Neglect (.91), while the internal consistency for Physical Neglect (.61) was acceptable. These values are comparable to published data on a 24-item Dutch CTQ-SF administered to community and psychiatric populations (Cronbach’s *α* = .63-.95; 28). Note that this 24-item version dropped the “molested” item, resulting in 4 rather than 5 items for sexual abuse; while “molested” clearly refers to sexually abusive behavior in English, the translated Dutch word does not necessarily have this sexual connotation. Nevertheless, to be comparable to other studies using the English CTQ-SF (e.g., 9, 35) we kept item 24 in the present study (and the good internal consistency of the Sexual Abuse subscale suggests this was not a problem).

***Common Somatic Symptoms***

The Patient Health Questionnaire (PHQ-15) is a checklist of 15 somatic symptoms that captures >90% of all physical complaints in outpatient settings (36). Each symptom is rated on a three-point scale (0 = *not bothered at all*, 1 = *bothered a little*, and 2 = *bothered a lot*). Cut-off scores indicate low (≤ 5), medium (≤ 10), and high (≥ 15) somatic symptom severity. Although respondents are usually asked about the past month, the HND study administered five versions of the PHQ-15 (2013-2018) to examine symptom burden over various time intervals (past 24h, 1 or 2 or 4 weeks, or 3 months). A previous HND study on the PHQ showed that scores increased with longer time frames and identified four weeks as optimal to capture clinically relevant subjective somatic symptom burden (28). Nonetheless, in this study we selected the 24h time frame because this version was completed by 1842 participants (vs. 300 for the 4-week version), which means a large sample and also the benefit of lower recall bias (37). A disadvantage of the 24h time frame is a lower likelihood of capturing participants’ episodic somatic symptoms and general symptom burden (28).

The PHQ-15 demonstrated a good internal consistency (*α* = .80) in previous studies (36) and was acceptable in our study (*α* = .75).

***Relevant EMA items***

Momentary somatic symptoms were examined using the item “I experience physical discomfort” to which participants could respond using a Visual Analogue Scale (VAS) from 0 (*not at all*) to 100 (*very much*). Social company during the past 6-hour time interval was assessed with the item ‘Most of the time since the last measurement I was’ (1 = alone, 2 = in company).

If participants were alone, their preference for being in company was assessed with the item “I would rather have been with others”, rated on a VAS ranging from 0 (*No, preferably not*) to 100 (*Yes, certainly*). If participants were in company, their preference for being alone was assessed with the item “I would rather have been alone” rated on a VAS from 0 (*No, preferably not*) to 100 (*Yes, certainly)*. Besides, their appraisal of the social company was assessed with the item “I found my company predominantly …”, rated on a VAS ranging from 0 (*Very unpleasant*), via 50 (*Neutral)* to 100 (*Very pleasant*).

***Covariates***

Sociodemographic information on gender (0 = male, 1 = female), age, relationship status (0 = single, 1 = has a partner), and education level (0 = secondary education or lower, 1= tertiary education) was obtained, since these variables have previously been associated with childhood trauma (38) and somatic symptoms (39). Indeed, these variables were included as covariates in the analyses due to their correlation with the CTQ and PHQ and due to their distribution in the sample (see Table 1).

**Statistical Analysis**

Both analyses of covariance (ANCOVA) with Bonferroni correction and multiple regression analyses were performed to test our hypothesis of a positive association between childhood trauma and somatic symptoms in the cross-sectional data. To examine whether specific types of childhood trauma predicted somatic symptoms, we also regressed total PHQ symptom scores on all five trauma type scores. These analyses were performed in SPSS 26 (IBM Corp., Armonk, NY).

To examine the validity of the EMA measure, a first multilevel model included the somatic symptom score as a predictor of momentary physical discomfort. To test our hypothesis of a positive association between childhood trauma and somatic symptoms in the EMA data, a second multilevel model was fit to examine the association between childhood trauma (categorical or continuous total CTQ scores) and momentary physical discomfort. A third multilevel model showed whether the association between childhood trauma and momentary discomfort was moderated by social company. Continuous total CTQ scores were centered so as to facilitate interpretation of a significant trauma by company interaction term. All multilevel models were run using SAS 9.4 (SAS Institute, Cary, NC) and included the same four covariates.

A *p*-value of .05 was used to test for statistical significance. Standardized regression coefficients (betas) were used to indicate the direction of the association between the variables. Effect sizes were estimated from *F* or *t* statistics and corresponding (denominator) degrees of freedom and expressed as *d* values. Effect sizes for between-person associations are typically interpreted as small (.10-.20) to moderate (.20 to .30) to large in magnitude (40).

**Results**

**Sample description**

The characteristics of the cross-sectional sample are provided in Table 1. Women (*n* = 284) reported more somatic symptoms on the PHQ than men (*n* = 122, *r* = .21, *p* < .001) and older participants reported more trauma (*r* = .18, *p* < .001). PHQ scores of participants who completed the CTQ (*n* = 406) were comparable to CTQ non-completers (*n* = 1436, *t*_(1840)_ = 1.239, *p* = .22). Participants who completed the PHQ (vs. those who did not) typically reported lower CTQ scores (*n* = 406 vs. *n* = 1189, *t*_(1593)_ = 3.249, *p* = .001).

The characteristics of the longitudinal EMA sample are also provided in Table 1. Women (*n* = 242) reported more physical discomfort than men (*n* = 48, *r* = .17, *p* = .004) and more traumatized participants were less educated (*r* = -.14, *p* = .017) and less likely to be in a romantic relationship (*r* = -.12, *p* = .041). Participation versus non-participation in the EMA study was independent of childhood trauma scores (*n* = 290 vs. *n* = 1305, t_(1593)_ = 1.20, *p* = .23).

Bivariate correlations between the five trauma types are reported in Table S1, Supplemental Digital Content, http://links.lww.com/PSYMED/A927, and were found to be moderately to strongly positive.

**Association between childhood trauma and common somatic symptoms**

The ANCOVA revealed that overall childhood trauma was positively associated with somatic symptom levels (*F*_(3, 398)_ = 7.73, *p* < .001, *d* = 0.28). See Figure 1a. Simple comparisons of adjusted PHQ-15 scores between groups with different trauma levels revealed no significant differences between those with no/minimal versus low/moderate trauma (*p* = .15), but higher somatic burden among participants with no/minimal versus moderate/severe trauma (*p* = .017) and no/minimal versus severe/extreme trauma (*p* = .001). The low/moderate trauma group showed fewer somatic symptoms than the severe/extreme trauma group (*p* = .020), but the low/moderate versus moderate/severe groups reported comparable symptom levels (*p* = .76), as did the moderate/severe versus severe/extreme groups (*p* = .58).

Similar results were obtained with multiple regression (see Table 2, model 1a; *F*_(5, 400)_ = 9.07, *p* < .001, *R^2^* = .10, *d* = 0.30). In a further regression analysis (see Table 2, model 2a), the link between childhood trauma and somatic symptoms remained significant when entering trauma types instead of the total trauma score (*F*_(9,396)_ = 7.43, *p* < .001, *R^2^* = .14, *d* = 0.28), although only the effects of Sexual Abuse and Emotional Abuse were significant. As the Variance Inflation Factor (VIF) was below 2.47 for each of the CTQ subscales, multicollinearity was not a concern in this analysis.

**Childhood trauma and momentary discomfort**

A multilevel model that included CTQ severity scores showed that more childhood trauma was associated with more momentary discomfort (*F*_(3, 291)_ = 6.87, *p* < .001, *d* = 0.31), see Figure 1b. The none/minimal trauma group reported less discomfort than the moderate to severe trauma group (*t*_(291)_ = -4.24, *p* < .001). There were no significant differences between none/minimal and low to moderate trauma groups (*t*_(291)_ = -2.36, *p* = .11 after Bonferroni correction), nor between the none/minimal versus severe to extreme trauma groups (*t*_(291)_ = -2.03, *p* = .26). The low to moderate trauma group reported less physical discomfort than the moderate to severe trauma group (*t*_(291)_ = -2.68, *p* =.047). No significant differences in discomfort were observed between the low to moderate and severe to extreme trauma groups (*t*_(291)_ = -1.08, *p* = 1.00) nor between the moderate to severe and severe to extreme trauma groups (*t*_(291)_ = 0.55, *p* = 1.00).

The multilevel model with the (centered) continuous total CTQ score as predictor showed similar results (Table S2, model 1, Supplemental Digital Content, http://links.lww.com/PSYMED/A927). However, when entered as main effects instead of the total CTQ score, the trauma types did not significantly predict momentary physical discomfort (Table S2, model 2). Nonetheless, when all interaction terms were also entered, the main effects of emotional abuse (*t*_(290)_ = 2.66, *p* = .008) and physical neglect (*t*_(290)_ = 2.77, *p* = .006) became significant. The 5-way interaction term including all trauma types was also significant (*t*_(290)_ = 2.50, *p* = .013), which suggests that momentary physical discomfort was partly explained by the cumulative effects of different types of childhood trauma.

**The role of social company in the association between childhood trauma and momentary physical discomfort**

This model confirmed the positive association between childhood trauma (centered total CTQ scores) and physical discomfort (*b* = 0.34, *F*_(1,291)_ = 14.37, *p* < .001, *d* = 0.44). There was also a main effect of interpersonal context, indicating that participants reported less discomfort after having been in social company (*b* = 0.66, *F*_(1,22*10_^3^_)_ = 5.38, *p* = .020, *d* = 0.03). However, the trauma by context interaction term was not significant, indicating that recent social company did not significantly moderate the link between trauma and momentary physical discomfort (*F*_(1, 22*103)_ = 0.01, *p* = .92).

Nonetheless, when we entered the CTQ subscale scores instead of the CTQ total scores, the physical neglect by interpersonal context interaction was significant (*F*_(1, 22*103)_ = 4.90, *p* = .027). Follow-up tests showed that although physical discomfort was not significantly associated with social company among individuals with less physical neglect (*b* = 0.31, *t*_(22*103)_ = 0.98, *p* = .33, *d* = 0.01), individuals with more physical neglect reported more physical discomfort when they were alone versus in company (*b* = 1.01, *t*_(22*103)_ = 3.12, *p* = .002, *d* = 0.04).

**Additional findings in the EMA data**

More childhood trauma predicted a preference for being alone when in company (*b* = 0.16, *t*_(291)_ = 2.34, *p* = .020), but did not significantly predict participants’ appraisal of their social company (*b* = -0.08, *t*_(291)_ = -1.65, *p* = .10). Childhood trauma was also not significantly associated with a preference for company when alone (*b* = -0.01, *t*_(290)_ = -0.11, *p* = .91).

Subsequently, we explored whether participants’ preferences and appraisal moderated the link between their childhood trauma and somatic symptoms. When participants were in company, they reported more physical discomfort when they had been more traumatized (*F*_(1,290)_ = 14.17, *p* < 0.001, *d* = 0.44), and also when they more strongly preferred to be alone (within-person centered; *F*_(1,14*103)_ = 165.49, *p* < .0001, *d* = 0.22), but there was no significant interaction effect (*F*_(1, 14*103)_ = 1.14, *p* = .29). Also when participants were in company, those with more negative appraisals of their company reported more physical discomfort (*F*_(1,14*103)_ = 77.76, *p* < .0001, *d* = 0.15), but there was no significant interaction between trauma and appraisal (*F*_(1, 14*103)_ = 0.01, *p* = .94).

When participants were alone, they also reported more physical discomfort when they had been traumatized more (*F*_(1,289)_ = 13.78, *p* < .001, *d* = 0.44). However, their preference for company played no role in their physical comfort levels (*F*_(1,7027)_ = 0.01, *p* = .93, *d* = 0.00), and there was no significant interaction between trauma and preferring company (*F*_(1,7027)_ = 0.28, *p* = .60).

**Discussion**

We examined the link between childhood trauma and adult somatic symptoms in a Dutch community sample. We expected and indeed observed more somatic symptom burden among more traumatized participants, both in terms of common somatic symptoms (assessed using the PHQ-15) and in terms of momentary physical discomfort (assessed repeatedly over 30 days using EMA).

Additionally, using the PHQ-15, we found associations between *specific* childhood trauma types and somatic symptoms. The link between childhood trauma and adult somatic burden was driven by emotional and sexual abuse. We observed no significant association between physical abuse or neglect or emotional neglect and the common somatic symptoms.

In the EMA data, however, none of the specific trauma types were significantly associated with physical discomfort. Moreover, we found no substantial role for recent social company in explaining the link between past trauma and momentary discomfort, although participants with a history of childhood physical neglect reported more physical discomfort when they were alone (vs. in company). These results are now discussed in more detail below.

**Contributions to the literature**

Our present results align with past studies that connect childhood trauma to various adult somatic symptoms (e.g., cardiovascular disease, gastrointestinal symptoms (41, 42)). We observed a dose-response pattern that differentiated between participants with no to moderate versus moderate to extreme childhood trauma, see Figure 1, which is in keeping with the literature showing that childhood trauma predicts the risk of adult physical health problems in a strong and cumulative fashion (4,35). Especially past sexual abuse showed strong associations with current somatic symptoms (in line with 43,44), and we also observed a clear link with emotional abuse (in line with 9). This latter finding is particularly worrisome given the high prevalence of emotional abuse in community surveys (29% in Europe (3)). Similarly, up to ~20% of the HND participants report moderate to severe emotional abuse.

Childhood physical abuse played a negligible role in explaining differences in adult somatic symptoms, which is not fully consistent with previous work (25,45). One possible explanation for this may be our community sample in which participants were not selected for physical abuse, and moderate to severe physical abuse was rare in comparison to past research (4% vs. 12%, respectively (45)). Note that physical abuse showed no incremental association with somatic symptom burden *over* emotional and sexual abuse, even though we observed substantial overlap in trauma types.

The present study also did not support previously reported associations between childhood emotional and physical neglect on the one hand and adult physical health (9) or health conditions such as diabetes, cancer, and hypertension (35) on the other hand. Our study differs from this previous work by using a mixed-gender community sample rather than an all-female primary care sample (9) and by using a common somatic symptom checklist covering the past 24h rather than assessing various health conditions (35). Besides, it is worth noting that some past research used a larger sample (*n* = 2510; 35) which increased statistical power and might have led to different outcomes.

Overall, our results suggest that the effects of childhood abuse versus neglect may differ. Child abuse may be particularly detrimental to lifelong health due to the *intentionality* of caregivers’ behaviors toward the child (i.e., acts of commission). Indeed, sexual and emotional abuse have been shown to have direct effects on adult physical health outcomes due to children’s stress reactions to the abuse (46). In comparison, neglect refers to the failure of caregivers to meet the child’s needs (i.e., acts of omission). As the latter is more passive, it may have long-term effects that are more indirect (47). However, other studies highlight that neglect can be as damaging to a child as abuse (9). Thus, our results should be interpreted with caution, at least until they are replicated.

Although there was no overall association between childhood physical neglect and adult somatic symptoms in the cross-sectional data nor in the EMA data, additional analyses in the EMA data revealed that recent social company moderated the association between childhood physical neglect and adult physical discomfort. Individuals who experienced more physical neglect reported less physical discomfort after periods spent in company, compared to periods spent alone. When in company, people may shift their attention to other people which might lead them to stop monitoring their own body, which they may do when alone, particularly if being alone is associated with more negative affect (48). Also, physically neglected adults were not taken care of when they were children. Consequently, they may not have learned to disclose their physical discomfort while growing up, and thus continue to internalize their physical symptoms in adulthood.

Social company did not significantly moderate the link between other childhood trauma types or total trauma scores and adult physical discomfort. This may indicate that participants with past traumatic experiences, which are often interpersonal in nature, learned to feel comfortable and safe around other people (being no longer a source or reminder of trauma). Indeed, more traumatized individuals did not rate their company as more unpleasant.

Nonetheless, more traumatized individuals preferred to be alone more when in company, in line with more disengaged and avoidant coping (49), and being alone was associated with more physical discomfort. This finding connects to past studies showing associations between childhood trauma and symptoms of social anxiety (e.g., avoidance, distress in social situations (50,51)), which in turn has been positively associated with somatic symptoms (52).

**Strengths and limitations**

The present study has several strengths, including the use of a community sample, assessment of different types of childhood trauma, and multiple methods to assess somatic symptoms, including EMA. Also, a consideration for the interpersonal context in which somatic symptoms might occur (i.e., social company) and the appraisal of this context was novel.

The study is limited by several weaknesses that are, however, common to studies of childhood trauma and adult physical health. Firstly, the CTQ and PHQ-15 are single-occasion snapshot measures. These measures are potentially colored by under- or over-reporting (e.g., due to current psychopathology, which we did not consider as a potential confounder of the observed associations) and cannot prove a causal relation between childhood abuse and adult somatic symptoms.

Secondly, we used a 24-h time frame for the PHQ-15 even though it does not capture somatic symptom burden as well as the original four-week time frame (28). Certain somatic symptoms, such as palpitations, fluctuate mostly over longer time periods. Thus, the association between childhood trauma and somatic symptoms might be confounded by the time frame used for the PHQ-15.

Thirdly, while the CTQ has been well-validated (32), asking adults to self-report on childhood events will always be influenced by retrospection (2,53). Most relevant here is our finding that older participants reported more childhood trauma. This appears to be in line with the general decline in trauma exposure over the past decades (e.g., in terms of rates of sexual and physical abuse (6)) but may also indicate an over-reporting among older adults or under-reporting or a memory bias in younger adults, possibly due to denial and embarrassment (54) or to age-related differences in psychopathology. Relatedly, the CTQ does not take into consideration that the frequency and duration of past abuse and neglect can vary greatly.

Fourthly, the HND sample may not be optimally representative of the general Dutch population, given the crowdsourcing approach used to recruit participants. Presumably, self-selection bias helps explain the relatively high educational levels and high proportion of women in the sample (30). Compared to the Dutch population, participants were also more likely to be in a romantic relationship, and, in the cross-sectional study, they were on average older. Our preliminary analyses showed additionally that people with lower CTQ scores were more likely to also complete the PHQ. This might be due to various factors, for example less current psychopathology in individuals with less past trauma. Thus, the cross-sectional results, obtained in participants who completed both the CTQ and the PHQ, might not generalize to all participants with CTQ scores.

Fifthly, equidistant time intervals for the individual EMA assessments might have compromised the quality of the data; predictable invitations to complete the EMA questionnaire might over time have increased participants’ anticipation and awareness. However, more unpredictable invitations might have led to more missing data, which already was substantial with >50% of participants being excluded as they were non-compliant. At the same time, this is not unusual for EMA protocols such as the one employed in the HND project, and previous research suggests that demographics, clinical diagnosis, personality, or number of daily measures, do not typically impact the EMA completion rate too much (55).

**Future research**

Future work could extend the present EMA approach to assess specific somatic symptoms rather than overall physical discomfort, which is a broad term that potentially allows for multiple interpretations. Perhaps more importantly, future EMA studies might dive deeper into the interpersonal factors that could potentially either increase or decrease somatic symptom levels among adults who experienced childhood abuse and neglect (56). Indeed, future work might benefit from adopting an event-contingent recording method, focusing on social interactions as the events of interest (57, 58), instead of using time-contingent recording like in the HND study. For example, the use of record forms similar to those used in a previous EMA study on blood pressure (58), which involved participants recording physical symptoms in response to social interactions with various other people, would be useful to extend the current findings.

Intimate relationships may involve the most difficult social interactions for adults who have experienced childhood abuse and neglect, as their expectations of current relationships are often based on past, negative interactions with primary caregivers in childhood (59). Thus, it may be worth examining with whom people reporting somatic symptoms were recently in contact (e.g., partner, close friend, family, a stranger) and not merely whether they were in contact with others. This idea is supported by the finding that childhood trauma is common in individuals diagnosed with mental disorders (60), which are often characterized by interpersonal problems. Signs of rejection (e.g., a partner is perceived as being quarrelsome) may activate attachment vulnerabilities and can trigger negative affect. In turn, negative affect can facilitate quarrelsome behavior, which might create a cycle of relationship problems (61).

**Conclusion**

This study showed that Dutch adults who reported more childhood trauma report a higher somatic symptom burden. We identified past sexual abuse and emotional abuse as potential key drivers of current somatic symptoms. Our results appear to support the idea that effects of childhood trauma on the body continue long after the abuse ends and make people more vulnerable to physical complaints and illness later in life. Health care professionals need to be aware how childhood trauma affects physical health across the lifespan, in order to reduce suffering and health care costs for the individual and society.

To the best of our knowledge this was the first study that explored, in line with the inherently interpersonal nature of much of childhood abuse and neglect, whether momentary physical discomfort among adults with childhood trauma is influenced by interpersonal factors. Our results provide preliminary evidence for the idea that that the presence of other people might moderate the association between at least some form of childhood trauma and adult physical discomfort. Future studies shall help determine how exactly interpersonal factors may shape the link between childhood trauma and adult somatic symptoms.

*Acknowledgement:* *We wish to thank all HowNutsAreTheDutch (HND) participants for their contribution.*

**References**

1. World Health Organization. Preventing child maltreatment: a guide to taking action and generating evidence; 2006.
2. Stoltenborgh M, Bakermans-Kranenburg MJ., Alink LRA, van IJzendoorn MH. The Prevalence of Child Maltreatment across the Globe: Review of a Series of Meta-Analyses. Child Abuse Review 2014;24(1):37–50. https://doi.org/10.1002/car.2353
3. Sethi D, Bellis M, Hughes K, Gilbert R, Mitis F, Galea G. European report on preventing child maltreatment. WHO Regional Office for Europe, Copenhagen. 2013. <https://apps.who.int/iris/handle/10665/326375>
4. Felitti VJ, Anda RF, Nordenberg D, Williamson DF, Spitz, AM, Edwards V et al. Relationship of childhood abuse and household dysfunction to many of the leading causes of death in adults: The Adverse Childhood Experiences (ACE) Study. American Journal of Preventive Medicine 1998;14(4):245-258. <https://doi.org/10.1016/S0749-3797(98)00017-8>
5. Norman RE, Byambaa M, De R, Butchart A. The long-term health consequences of child physical abuse, emotional abuse, and neglect: a systematic review and meta-analysis. PLoS Medicine 2012; 9(11). <https://doi.org/10.1371/journal.pmed.1001349>
6. World Health Organization. Global status report on preventing violence against children; 2020.
7. Green, CR, Flowe-Valencia H, Rosenblum L, Trait AR. The Role of Childhood and adulthood abuse among women presenting for chronic pain management. Clinical Journal of pain 2001; 17: 359-364.
8. Benedixen M, Muus KM, Schei B. The Impact of child sexual abuse: A case study of a random sample of Norwegian students. Child Abuse & Neglect 1994; 16: 837-847.
9. Spertus IL, Yehuda R, Wong CM, Halligan S, Seremetis SV. Childhood emotional abuse and neglect as predictors of psychological and physical symptoms in women presenting to a primary care practice. Child Abuse & Neglect 2003;27(11):1247-1258. <https://doi.org/10.1016/j.chiabu.2003.05.001>
10. Afari N, Ahumada SM, Wright LJ, Mostoufi S, Golnari G, Reis V, Cuneo JG. Psychological trauma and functional somatic syndromes: a systematic review and meta-analysis. Psychosomatic medicine 2014; 76(1), 2. https://doi.org/10.1097%2FPSY.0000000000000010s
11. Danese A, McEwen BS. Adverse childhood experiences, allostasis, allostatic load, and age-related disease. Physiology & Behavior 2012;106(1): 29-39. <https://doi.org/10.1016/j.physbeh.2011.08.019>
12. Heim C, Newport DJ, Heit S, Graham YP, Wilcox M, Bonsall R et al. Pituitary-adrenal and autonomic responses to stress in women after sexual and physical abuse in childhood. Journal of the American Medical Association 2000;284: 592–597. <http://doi.org/10.1001/jama.284.5.592>
13. Schury K, Kolassa I. Biological memory of childhood maltreatment: current knowledge and recommendations for future research. Annals of the New York Academy of Sciences 2012;1262(1):93-100. <https://doi.org/10.1111/j.1749-6632.2012.06617.x>
14. Schenk HM, Jeronimus BF, van der Krieke L, Bos EH, de Jonge P, Rosmalen JG. Associations of positive affect and negative affect with allostatic load: a lifelines cohort study. Psychosomatic medicine 2018;80(2):160-166. <https://doi.org/10.1097/PSY.0000000000000546>
15. Sedlak AJ, Mettenburg J, Basena M, Petta I, McPherson K, Greene A et al. Fourth National Incidence Study of Child Abuse and Neglect (NIS–4): Report to Congress. Washington, DC: U.S. Department of Health and Human Services, Administration for Children and Families. 2010.
16. Bandura A. Social learning theory. Prentice-Hall; 1977.
17. Bowlby J. Attachment and loss. Basic Books; 1969
18. Messman-Moore T, Coates AA. The impact of childhood psychological abuse on adult interpersonal conflict: The role of early maladaptive schemas and patterns of interpersonal behavior. Journal of Emotional Abuse 2007;7(2):75-92. <https://doi.org/10.1300/J135v07n02_05>
19. Wheeler NJ, Daire AP, Barden SM, Carlson RG. Relationship distress as a mediator of adverse childhood experiences and health: Implications for clinical practice with economically vulnerable racial and ethnic minorities. Family Process 1019;58(4):1003-1021. <https://doi.org/10.1111/famp.12392>
20. Brewin CR, Andrews B, Valentine JD. Meta-analysis of risk factors for posttraumatic stress disorder in trauma-exposed adults. Journal of Consulting and Clinical Psychology 2000;68(5):748-766. <https://doi.org/10.1037/0022-006X.68.5.748>
21. Kimerling R, Calhoun KS. Somatic Symptoms, Social Support, and Treatment Seeking among Sexual Assault Victims. Journal of Consulting and Clinical Psychology 1994;62(2):333-40. [http://doi.org/10.1037//0022-006x.62.2.333](http://doi.org/10.1037/0022-006x.62.2.333)
22. Cohen S, Wills TA. Stress, social support, and the buffering hypothesis. Psychological Bulletin 1985;98(2):310-57. <https://doi.org/10.1037/0033-2909.98.2.310>
23. Suglia SF, Koenen KC, Boynton-Jarrett R, Chan PS, Clark CJ, Danese A et al. American Heart Association Council on Epidemiology and Prevention, Council on Cardiovascular Disease in the Young, Council on Functional Genomics and Translational Biology, . . . and Council on Quality of Care and, O. Childhood and Adolescent Adversity and Cardiometabolic Outcomes: A Scientific Statement From the American Heart Association. Circulation 2018;137(5):e15-e28. <https://doi.org/10.1161/CIR.0000000000000536>
24. Henningsen P, Zipfel S, Sattel H, Creed F. Management of functional somatic syndromes and bodily distress. Psychotherapy and Psychosomatics 2018; 87(1): 12-31. https://doi.org/10.1159/000484413
25. Greenfield EA. Child abuse as a life-course social determinant of adult health. Maturitas 2010;66(1):51-55. <https://doi.org/10.1016/j.maturitas.2010.02.002>
26. Sachs-Ericsson N, Blazer D, Plant EA, Arnow B. Childhood Sexual and Physical Abuse and the 1-Year Prevalence of Medical Problems in the National Comorbidity Survey. Health Psychology 2005;24(1):32-40. <https://doi.org/10.1037/0278-6133.24.1.32>
27. Conner TS, Barrett LF, Tugade MM, Tennen H. Idiographic personality: The theory and practice of experience sampling. In Robins RW, Fraley RC, Krueger RF. Handbook of Research Methods in Personality Psychology 2007:79-96.
28. Joustra ML, Janssens KAM, Schenk HM, Rosmalen JGM. The four week time frame for somatic symptom questionnaires reflects subjective symptom burden best. Journal of Psychosomatic Research 2018;104:16-21. <https://doi.org/10.1016/j.jpsychores.2017.11.006>
29. Myin-Germeys I, Kuppens P. The Open Handbook of Experience Sampling Methodology: A step-by-step guide to designing, conducting, and analyzing ESM studies. Center for Research on Experience Sampling and Ambulatory Methods Leuven (REAL); 2021.
30. Van Der Krieke L, Jeronimus BF, Blaauw FJ, Wanders RBK, Emerencia AC, Schenk HM et al. HowNutsAreTheDutch ((HoeGekIsNL): A crowdsourcing study of mental symptoms and strengths. International Journal of Methods in Psychiatric Research 2016;25(2):123-144. <https://doi.org/10.1002/mpr.1495>
31. van der Krieke L, Blaauw FJ, Emerencia AC, Schenk HM, Slaets JP, Bos EH, et al. Temporal dynamics of health and well-being: A crowdsourcing approach to momentary assessments and automated generation of personalized feedback. Psychosomatic medicine 2017;79(2):213-223. <https://doi.org/10.1097/PSY.0000000000000378>
32. Bernstein DP, Stein JA, Newcomb MD, Walker E, Pogge D, Ahluvalia T et al. Development and validation of a brief screening version of the Childhood Trauma Questionnaire. Child Abuse & Neglect 2003;27(2):169-190. <https://doi.org/10.1016/S0145-2134(02)00541-0>
33. Thombs BD, Bernstein DP, Lobbestael J, Arntz A. A validation study of the Dutch Childhood Trauma Questionnaire-Short Form: Factor structure, reliability, and known-groups validity. Child Abuse & Neglect 2009;33(8):518-523. <https://doi.org/10.1016/j.chiabu.2009.03.001>
34. Bernstein DP & Fink L. Childhood Trauma Questionnaire: A retrospective self-report manual San Antonio, TX: The Psychological Corporation 1998.
35. Clemens V, Huber-Lang M, Plener PL, Brähler E, Brown RC, Fegert JM. Association of child maltreatment subtypes and long-term physical health in a German representative sample. European Journal of Psychotraumatology 2018;9. <https://doi.org/10.1080/20008198.2018.1510278>
36. Kroenke K, Spitzer RL, Williams JBW. The PHQ-15: Validity of a new measure for evaluating the severity of somatic symptoms. Psychosomatic Medicine 2002;64(2):258-266. <https://doi.org/10.1097/00006842-200203000-00008>
37. Broderick JE, Schwartz JE, Vikingstad G, Pribbernow M, Grossman S, Stone AA. The accuracy of pain and fatigue items across different reporting periods. Pain. 2008; 30;139(1):146-157. doi: 10.1016/j.pain.2008.03.024.
38. Witt A, Brown RC, Plener PL, Brähler E, Fegert JM. Child maltreatment in Germany: Prevalence rates in the general population. Child and Adolescent Psychiatry and Mental Health 2017;11. <https://doi.org/10.1186/s13034-017-0185-0>
39. Creed FH, Davies I, Jackson J, Littlewood A, Chew-Graham C, Tomenson B, Macfarlane G, Barsky A, Katon W, McBeth J. The epidemiology of multiple somatic symptoms. Journal of Psychosomatic Research 2012;72(4): 311-317. <https://doi.org/10.1016/j.jpsychores.2012.01.009>
40. Cohen J. Statistical power analysis for the behavioral sciences. 2nd ed. Hillside, NJ: Lawrence Erlbaum Associates; 1988.
41. Beilharz JE, Paterson M, Fatt S, Wilson C, Burton A, Cvejic E et al. The impact of childhood trauma on psychosocial functioning and physical health in a non-clinical community sample of young adults. Australian and New Zealand Journal of Psychiatry 2020;54(2):185-194. <https://doi.org/10.1177/0004867419881206>
42. Wegman HL, Steltler C. A meta-analytic review of the effects of childhood abuse on medical outcomes in adulthood. Psychosomatic Medicine 2009;71(8):805-812. <https://doi.org/10.1097/PSY.0b013e3181bb2b46>
43. Irish L, Kobayashi I, Delahanty DL. Long-term physical health consequences of childhood sexual abuse: A meta-analytic review. Journal of Pediatric Psychology 2010;35(5):450-461. <https://doi.org/10.1093/jpepsy/jsp118>
44. Kamiya Y, Timonen V, Kenny RA. The impact of childhood sexual abuse on the mental and physical health, and healthcare utilization of older adults. International psychogeriatrics 2016;28(3):415–422. <https://doi.org/10.1017/S1041610215001672>
45. Springer KW, Sheridan J, Kuo D, Carnes M. Long-term physical and mental health consequences of childhood physical abuse: Results from a large population-based sample of men and women. Child Abuse & Neglect 2007;31(5):517-530. <https://doi.org/10.1016/j.chiabu.2007.01.003>
46. Rueness J, Myhre MC, Strøm IF, Wentzel-Larsen T, Dyb G, Thoresen S. The mediating role of posttraumatic stress reactions in the relationship between child abuse and physical health complaints in adolescence and young adulthood. European Journal of Psychotraumatology 2019;10(1). <https://doi.org/10.1080/20008198.2019.1608719>
47. Leeb RT, Paulozzzi L, Melanson C, Simon T, Arias I. Child maltreatment surveillance. Uniform definitions for public health and recommended data elements. Atlanta: Centers for Disease Control and Prevention; 2008.
48. Gendolla GHE, Abele AE, Andrei A, Spurk D, Richter M. Negative Mood, Self-Focused Attention, and the Experience of Physical Symptoms: The Joint Impact Hypothesis. Emotion 2005;5(2):131–144. [http://doi: 10.1037/1528-3542.5.2.131](http://doi:%2010.1037/1528-3542.5.2.131)
49. Griffing S, Lewis CS, Chu M, Sage R, Jospitre T, Madry L, Primm BJ. The Process of coping with domestic violence in adult survivors of childhood sexual abuse. Journal of Child Sexual Abuse 2006; 15(2): 23-41.
50. Feerick MM, Snow KL. The relationships between childhood sexual abuse, social anxiety, and symptoms of posttraumatic stress disorder in women. Journal of Family Violence, 2005;20(6). <http://doi.org/10.1007/s10896-005-7802-z>
51. Kuo JR, Goldin PR, Werner K, Heimberg RG, Gross JJ. Childhood trauma and current psychological functioning in adults with social anxiety disorder. Journal of Anxiety Disorders 2011;25(4):467-473. <https://doi.org/10.1016/j.janxdis.2010.11.011.>
52. May AC, Rudy BM, Davis TE, Jenkins WS, Reuther ET, Whiting SE. Somatic symptoms in those with performance and interaction anxiety. Journal of Health Psychology 2014;19(11):1400–1409. <https://doi-org/10.1177/1359105313490773>
53. Hardt J & Rutter M. Validity of adult retrospective reports of adverse childhood experiences: review of the evidence. Journal of Child Psychology and Psychiatry 2004; 4(2): 260-273.
54. Fergusson DM, Horwood LJ, Woodward LJ. The stability of child abuse reports: A longitudinal study of the reporting behaviour of young adults. Psychological Medicine 2000;30(3):529-544. <https://doi.org/10.1017/S0033291799002111>
55. Soyster PD, Bosley HG, Reeves JW, Altman AD, Fisher AJ. Evidence for the Feasibility of Person-Specific Ecological Momentary Assessment Across Diverse Populations and Study Designs. Journal for person-oriented research 2019;5(2):53–64. <https://doi.org/10.17505/jpor.2019.06>
56. Masten AS, Lucke CM, Nelson KM, Stallworthy IC. Resilience in Development and Psychopathology: Multisystem Perspectives. Annual Review of Clinical Psychology 2021;17(1):521–549. <https://doi.org/10.1146/annurev-clinpsy-081219-120307>
57. D'Antono B, Ditto B, Moskowitz DS, Rios N. Interpersonal behavior and resting blood pressure in college women: a daily monitoring study. Journal of psychosomatic research 2001;50(6):309–318.<https://doi.org/10.1016/s0022-3999(01)00199-4>
58. Moskowitz DS, Russell JJ, Sadikaj G, Sutton R. Measuring people intensively. Canadian Psychology/Psychologie Canadienne 2009;50(3):131-140. <https://doi.org/10.1037/a0016625>
59. Colman RA, Widom CS. Childhood abuse and neglect and adult intimate relationships: A prospective study. Child Abuse & Neglect 2004;28(11):1133-1151. <https://doi.org/10.1016/j.chiabu.2004.02.005>
60. Carr CP, Martins CM, Stingel AM, Lemgruber VB, Juruena MF. The role of early life stress in adult psychiatric disorders: a systematic review according to childhood trauma subtypes. The Journal of nervous and mental disease 2013;201(12):1007–1020. <https://doi.org/10.1097/NMD.0000000000000049>
61. Sadikaj G, Moskowitz DS, Russell JJ, Zuroff, DC, Paris J. Quarrelsome behavior in borderline personality disorder: influence of behavioral and affective reactivity to perceptions of others. Journal of abnormal psychology 2013;122(1):195–207. <https://doi.org/10.1037/a0030871>

**Figure caption**

Figure 1.
a. Adjusted means and standard deviations of somatic symptom burden (PHQ-15) among childhood trauma (CTQ) severity groups (N= 406).

*b.* Adjusted means and standard deviations of momentary physical discomfort (EMA) among childhood trauma (CTQ) severity groups (N= 290).

Lower physical discomfort scores in the severe to extreme trauma group than in the moderate to severe trauma group probably reflect the small number of participants in the former group. However, alternative explanations remain possible, such as a non-linear association between trauma and physical discomfort, via physical decoupling in the latter group.

CTQ-SF = Childhood Trauma Questionnaire Short Form. PHQ-15 = Patient Health Questionnaire. SD = standard deviation.
